# Integrating Key User Characteristics in User-Centered Design of Digital Support Systems for Seniors’ Physical Activity Interventions to Prevent Falls: Protocol for a Usability Study

**DOI:** 10.2196/20061

**Published:** 2020-12-21

**Authors:** Åsa Revenäs, Ann-Christin Johansson, Maria Ehn

**Affiliations:** 1 Center for Clinical Research Region Västmanland-Uppsala University Hospital of Västmanland Västerås Västerås Sweden; 2 School of Health, Care and Social Welfare Mälardalen University Västerås Sweden; 3 School of Innovation, Design and Engineering Mälardalen University Västerås Sweden

**Keywords:** eHealth, mobile health, internet-based interventions, physical activity, exercise, older adults, gender, user feedback, user involvement, user-centered design

## Abstract

**Background:**

The goal of user-centered design (UCD) is to understand the users’ perspective and to use that knowledge to shape more effective solutions. The UCD approach provides insight into users’ needs and requirements and thereby improves the design of the developed services. However, involving users in the development process does not guarantee that feedback from different subgroups of users will shape the development in ways that will make the solutions more useful for the entire target user population.

**Objective:**

The aim of this study was to describe a protocol for systematic analysis and prioritization of feedback from user subgroups in the usability testing of a digital motivation support for fall-preventive physical activity (PA) interventions in seniors (aged 65 years and older). This protocol can help researchers and developers to systematically exploit feedback from relevant user subgroups in UCD.

**Methods:**

Gender, PA level, and level of technology experience have been identified in the literature to influence users’ experience and use of digital support systems for fall-preventive PA interventions in seniors. These 3 key user characteristics were dichotomized and used to define 8 (ie, 2^3^) possible user subgroups. The presented method enables systematic tracking of the user subgroups’ contributions in iterative development. The method comprises (1) compilation of difficulties and deficiencies in the digital applications identified in usability testing, (2) clustering of the identified difficulties and deficiencies, and (3) prioritization of deficiencies to be rectified. Tracking user subgroup representation in the user feedback ensures that the development process is prioritized according to the needs of different subgroups. Mainly qualitative data collection methods are used.

**Results:**

A protocol was developed to ensure that feedback from users representing all possible variants of 3 selected key user characteristics (gender, PA level, and level of technology experience) is considered in the iterative usability testing of a digital support for seniors’ PA. The method was applied in iterative usability testing of two digital applications during spring/summer 2018. Results from the study on the users’ experiences and the iterative modification of the digital applications are expected to be published during 2021.

**Conclusions:**

Methods for systematic collection, analysis, and prioritization of feedback from user subgroups might be particularly important in heterogenous user groups (eg, seniors). This study can contribute to identifying and improving the understanding of potential differences between user subgroups of seniors in their use and experiences of digital support for fall-preventive PA interventions. This knowledge may be relevant for developing digital support systems that are appropriate, useful, and attractive to users and for enabling the design of digital support systems that target specific user subgroups (ie, tailoring of the support). The protocol needs to be further used and investigated in order to validate its potential value.

**International Registered Report Identifier (IRRID):**

RR1-10.2196/20061

## Introduction

### Background

The goal of user-centered design (UCD) is to understand the users’ perspective and to use that information to shape more effective solutions [1]. UCD gains access to users’ needs and requirements and thereby improves the solutions’ design and increases their functionality, usability, and quality [2,3]. UCD can reduce the users’ need for support, decrease development costs, and increase user satisfaction and safety [4]. However, involving users in the development process does not guarantee that feedback from different subgroups of users will equally shape the development in such a way that the developed solution becomes useful for the entire user population for which it was intended. This study describes a method that enables systematic tracking of feedback from user subgroups in usability testing of digital support for fall-preventive physical activity (PA) in seniors (adults aged 65 years and older). The aim of the method is to support the development of a digital support system that is useful for different subgroups of users.

### Digital Support for Improved Health in Seniors

Digital support systems—for example, mobile apps or web-based services—have the potential to strengthen and complement existing health care resources. There is evidence indicating that digital support can be effective in somatic care [5]. The internet is also an important source for acquiring disease-specific knowledge in chronic care management [6]. In 2019, approximately one-fifth of the population in the European Union was 65 years or older, and more than 5% of the population was 80 years and older [7]. The proportion of individuals aged 80 years and older is expected to more than double within the next 80 years. Furthermore, approximately 80% of the population aged 80 years and older suffers from at least one chronic condition [8]. Provision of digital support to improve health in the growing population of seniors is therefore increasingly important.

It is well established that PA has a positive impact on health and well-being. For example, physically active people have lower rates of lifestyle-related diseases (including coronary diseases, high blood pressure, type 2 diabetes, colon and breast cancer, and depression) and exhibit higher levels of functional health and better cognitive function [9]. Despite this knowledge, physical inactivity is an increasing global health burden [9].

### Involvement of Seniors in the Development of Digital Support Systems

User involvement in the development of digital support systems is an evolving area. A review on development of fall-detection systems concluded that seniors are never involved throughout the whole development process and seldom involved in the early and end stages of development [10]. The authors emphasize that early user involvement, focusing on the users’ needs and preferences, is important for improving the seniors’ level of technology acceptance. There is a great variation regarding which users are involved in UCD and how they are involved. Moreover, reporting on the procedures used for the selection and recruitment of users is often lacking [11].

Seniors’ interest in and use of the internet is associated with sociodemographic factors including gender, age, and socioeconomic status [12-15]. Moreover, seniors use the internet for different purposes: while some use it solely for practical things, such as financial purposes, searching for information, and emailing, others use it for additional purposes, such as gaming and social interaction [16]. In addition, seniors have different needs and preferences for digital health care support, partly driven by their own experiences with health care [13]. The described diversity in the older population challenges the UCD because involving certain users does not entail that their feedback will represent views of the entire target population. There is a need to increase the understanding of user subgroups’ needs and contribution in UCD processes in order to involve representatives of relevant stakeholders in the development.

### Purpose of the Study

The aim of this study was to describe a protocol for systematic analysis and prioritization of feedback from user subgroups in the usability testing of digital support systems to motivate fall-preventive PA in seniors. This protocol can help researchers and developers to systematically exploit feedback from relevant subgroups in UCD.

## Methods

### Overview of the UCD Process and Included Studies

**Figure 1:**
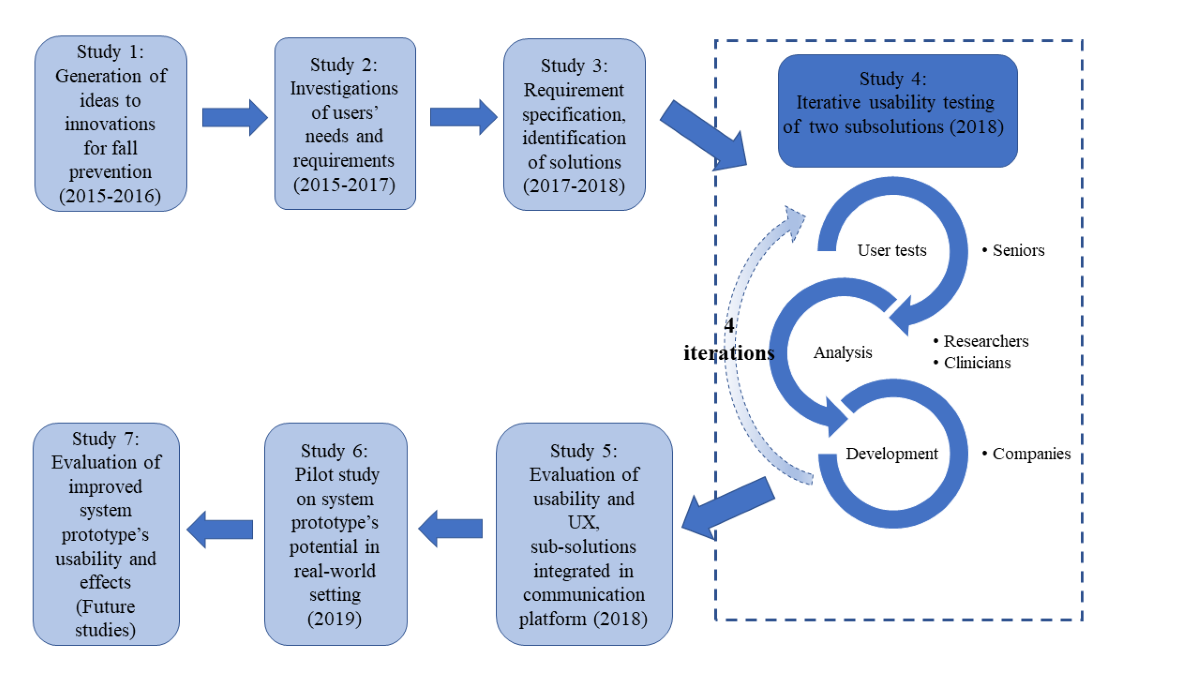
Overview of the user-centered design and the 7 studies (where study 7 represents several future studies) of the development of digital support for fall-preventive physical activity interventions in seniors. This protocol describes a method for systematic tracking of feedback from user subgroups used in study 4. UX: user experience.

This project is based on a UCD model [1] and the key principles described by Gulliksen et al [17], including early user involvement. An overview of the UCD process is presented in Figure 1. The protocol described in this article is used in study 4 (Figure 1).

### Identification of Key User Characteristics

Three key user characteristics have been identified in the literature to influence seniors’ experience of fall prevention, PA, and technology use: (1) gender, (2) PA level, and (3) level of technology experience. Gender was selected because a gender-based difference exists in PA behavior, falls incidence, and consequences from falls; men and women have different fall risk factors, both short- and long-term [18]. Compared to men, women are more prone to falling, sustain more fall-related injuries [19,20], and report fear of falling more frequently [21,22]. Men and women also differ in their exercise habits and reasons for exercise [23]: women are less likely than men to be regularly physically active [24] and tend to prefer different types of PA [25]. Gender has also been identified as crucial for understanding technology use [26]. PA level and level of technology experience were also selected because seniors with lower PA levels and less experience with technology might represent less engaged users of digital support services for PA. The 3 key user characteristics are integrated in the collection and analysis of user feedback.

### Definition of User Subgroups From Possible Combinations of Key User Characteristics

By dichotomizing the 3 key user characteristics, 8 (ie, 2^3^) possible user subgroups were defined. The participant’s PA level and level of technology experience were classified as “higher” or “lower,” respectively, according to the user’s self-reported PA level and level of technology experience in the questionnaire on user characteristics (Multimedia Appendix 1). Participants who reported spending at least 3 hours/week performing moderate-intensity activities during a regular week (questions 1 and 2, respectively, on PA level in Multimedia Appendix 1) were classified as having a higher PA level, while participants who reported spending less than 3 hours/week performing moderate-intensity activities were classified as having a lower PA level. The threshold was chosen based on the PA guidelines for the adult population, which recommend at least 150 minutes/week of moderate-intensity aerobic PA [9]. The cutoff for higher level of technology experience was set to using a mobile phone and/or computer/tablet often for purposes including calls, texting, emails, and surfing the internet, as reported by participants (questions 3 and 4 on level of technology experience in Multimedia Appendix 1). This cutoff was chosen to ensure that participants had experience in surfing the internet. The participant’s gender was classified as man or woman according to the participant’s self-reported gender in the questionnaire (Multimedia Appendix 1).

Examples of subgroups were men with a lower PA level and higher level of technology experience and women with a higher PA level and lower level of technology experience.

### Coding of Individual Users According to User Subgroups

The assignment of each participant to 1 of 8 subgroups was identified by analysis of the questionnaire on participant characteristics (Multimedia Appendix 1). Each participant’s user subgroup was visualized on all templates for processing and analysis of data generated by the specific participant.

### Usability Test Procedure

During the iterative testing (Figure 1, study 4), the participants tested and evaluated prototype versions of the digital support (2 applications) in 4 test cycles. Each participant performed the tests individually with a researcher. Qualitative and quantitative data on use and use experience were collected during and after the test sessions.

Table 1 presents an overview of the methods for data collection, processing, and analysis, and concretization of improvements applied in each test cycle. Steps 1 to 3 were included in the method for systematic tracking of user subgroups’ feedback and they are described in detail below.

**Table 1:** Overview of the data collection, processing, and analysis, as well as the concretization of improvements in the usability testing, highlighting the 3 steps included in the subgroup method.

Research phase	Description of actions and the 3 steps included in the subgroup method
Data collection	Multiple sources of mainly qualitative data on participants’ management and experiences of the applications: questionnaire on user characteristics (Multimedia Appendix 1) observation protocol (Multimedia Appendix 2) written interview and user rating documentation (Multimedia Appendix 3) audio recording
Data processing	Summary of observations and answers to rating and interview questions for each user Step 1: Identification of difficulties and deficiencies in the applications and the user subgroups experiencing them compilation of difficulties/deficiencies from data collected on participants’ experiences paper strips, containing 1 experienced difficulty/deficiency each, tagged with the participant’s user subgroup
Data analysis including prioritization of difficulties/deficiencies to be rectified/improved	Step 2: Clustering of difficulties and deficiencies thematic analysis, by sorting the paper-strips manually labeling themes to reflect the difficulty/deficiency tagged with an aggregation of user subgroups Step 3: Prioritization based on user subgroup representation, importance and impact
Concretization of improvements	Concretization of actions to be taken for prioritized improvements

#### Step 1: Identification of Difficulties and Deficiencies in the Applications and the User Subgroups Experiencing Them

**Figure 2:**
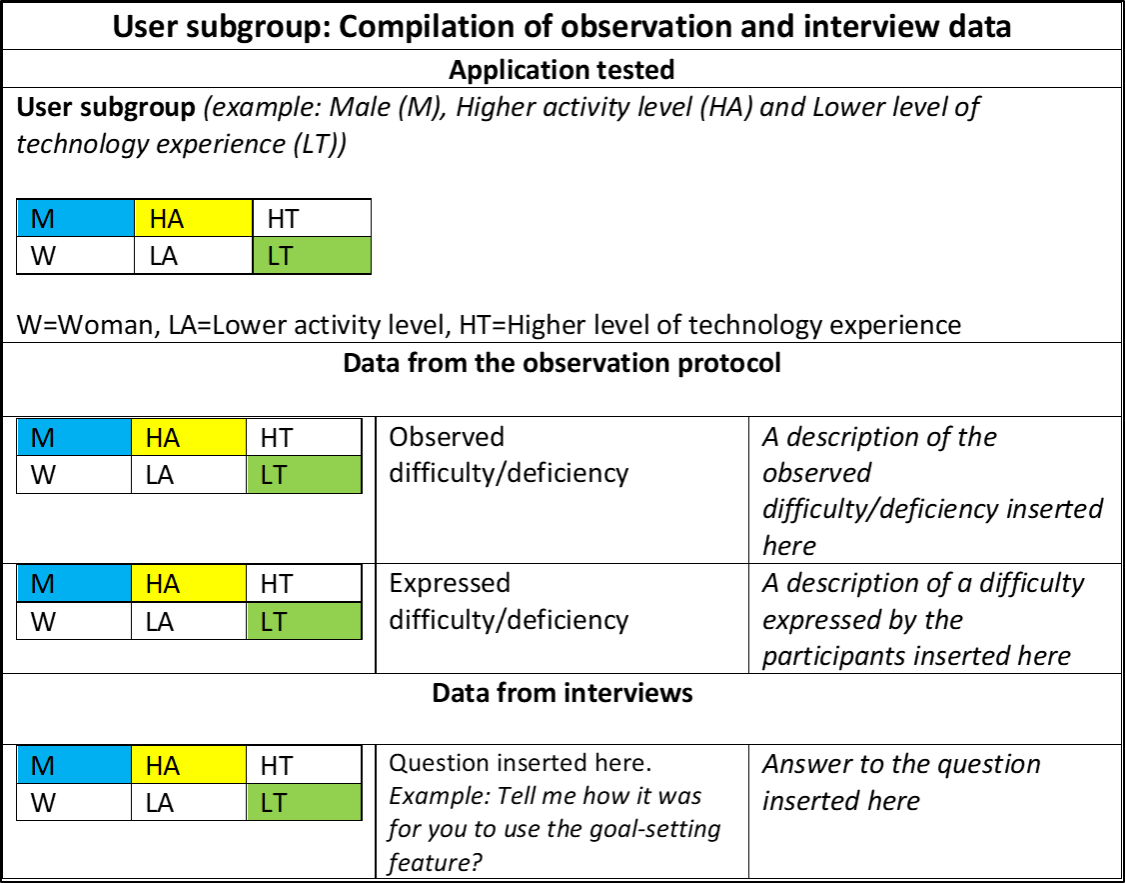
The template used in step 1 for the compilation of observation and interview data.

The data on difficulties/deficiencies in the applications collected during test sessions is compiled in a predefined template (Figure 2). In the template, each row documents one identified difficulty/deficiency tagged with the user subgroup of the participant (colored to improve visualization of different key characteristics). One template is completed for each participant. The template can be expanded by the addition of as many new rows as needed.

After compiling data on observed or expressed difficulty/deficiency for all participants, the templates are printed out on paper and cut into paper strips. Hence, each strip contains one observed/expressed difficulty/deficiency tagged with the participant’s user subgroup.

The main results from this step are paper strips containing observed/expressed difficulty/deficiency identified during the usability testing tagged with user subgroup.

#### Step 2: Clustering of Difficulties and Deficiencies

**Figure 3:**
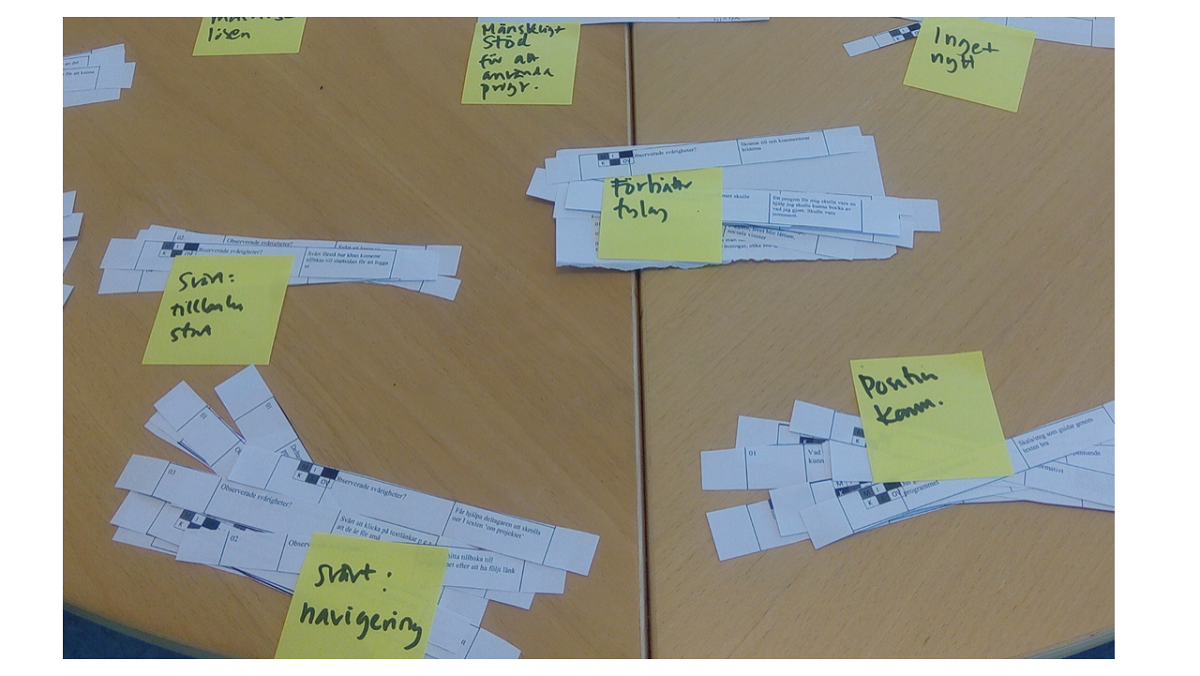
Thematic analysis and clustering of the paper strips. Each cluster is labeled according to the difficulties/deficiencies it reflects.

All paper strips from step 1 are analyzed qualitatively. Any qualitative method can be used, for example the thematic analysis described by Braun and Clarke [27]. The paper strips are sorted manually according to similarity in the difficulty or deficiency they reflect and subsequently labeled (Figure 3).

The main results from this step are themes describing deficiencies/difficulties in the digital applications tagged with an aggregation of user subgroups that all participants experiencing the difficulties belonged to (hereafter denoted “user subgroup aggregation”). For example, if participants from the two user subgroups “men with lower PA level and higher level of technology experience” and “women with higher PA level and higher level of technology experience” express the same deficiency, the resulting theme (ie, deficiency) is tagged with the user subgroup aggregation (ie, “men and women with lower and higher PA level and higher level of technology experience”).

#### Step 3: Prioritization Based on User Subgroup Aggregation, Importance, and Impact

Themes reflecting deficiencies/difficulties in the applications tagged with aggregated user subgroups (identified in step 2) are further grouped according to the feature they occur in (eg, navigation, goal setting, or feedback). All features with identified deficiencies/difficulties tagged with user subgroup aggregations are summarized in a prioritization template (Table 2).

Prioritization of difficulties/deficiencies to be improved or rectified prior to the next test cycle is based on the following aspects: (1) whether the user subgroup aggregations represent all or most of the 8 possible user subgroups (ie, if both men and women with higher and lower PA levels and levels of technology experience have experienced the deficiency); (2) whether the deficiency can be related to the participants’ ratings of user experience or perceived value for support in fall-preventive PA interventions of different features, if applicable (this can be interpreted as “performance” and “effort expectancy”, two constructs important for the acceptability and usability of technology according to the unified theory of acceptance and use of technology [28]); and (3) whether the deficiency has high impact on the solution’s purpose and usability. Previous research has suggested that issues that are both brought up by users and have a large impact on the overall purpose of a solution should be considered in development [29]. The researchers’ assessment of aspects (1) to (3) for each deficiency is documented in a prioritization template (Table 2).

The main result from this step is a list of prioritized difficulties/deficiencies to be improved before the next test cycle or saved for later.

**Table 2:** The template used in step 3 for prioritization of difficulties and deficiencies to be improved or rectified, including a mock result.

		User subgroup aggregation experiencing deficiency						Prioritization				When to be improved?		
Features	Deficiency	M^a^	W^b^	HA^c^	LA^d^	HT^e^	LT^f^		All user characteristics represented?	Related to low ratings?^g^	High impact on the solution?		Now	Later
Goal set	Concepts difficult to understand	✓	✓	✓	✓	✓	✓		Yes	No	Yes		✓	
	Difficult to set a realistic PA goal^h^	✓	✓	✓	✓	✓			No	No	Yes			✓

^a^M: man.

^b^W: woman.

^c^HA: higher activity level.

^d^LA: lower activity level.

^e^HT: higher level of technology experience.

^f^LT: lower level of technology experience.

^g^The participants’ answers to the rating questions, on a 100 mm visual analog scale (see Multimedia Appendix 3 for more details).

^h^PA: physical activity.

## Results

This paper describes a novel approach to systematically track user subgroups’ feedback in usability testing of digital support systems. The method will ensure that feedback from users representing all possible variants of 3 selected key user characteristics (gender, PA level, and level of technology experience) are shaping the iterative development of digital support systems. An overview of the key activities in the subgroup tracking method is summarized in Table 3.

**Table 3:** Presentation of main activities in the method for identifying key user characteristics and systematic tracking of user subgroup feedback.

Research phase	Description of main activities in the subgroup method
Preparation	Identification of key user characteristics based on previous research and/or theory relevant for the focus area of the digital support system Definition of user subgroups from possible combinations of key user characteristics dichotomization of the key user characteristics Coding of individual users according to user subgroup visualization of subgroup on the templates used for data collection, processing, and analysis
Data processing	Identification of difficulties and deficiencies in the applications and the user subgroups experiencing them compilation of difficulties/deficiencies from data collected on users’ experiences paper strips containing an experienced difficulty/deficiency tagged with the user’s subgroup
Data analysis	Clustering of difficulties and deficiencies thematic analysis, by sorting the paper strips manually labeling themes to reflect the difficulty/deficiency tagged with an aggregation of user subgroups Prioritization based on user subgroup aggregations, importance, and impact

The method was applied in a study approved by the regional ethics committee in Uppsala, Sweden (Dnr 2018/044). In this study, the method was used in usability testing of two digital applications to motivate PA in seniors during spring/summer 2018. Results from the study on the users’ experiences and the iterative modification of the digital applications are expected to be published in 2021. The study will provide further insights into the potential of this novel approach for ensuring that needs and preferences of different user subgroups are captured and considered in a UCD process. Moreover, the results are expected to contribute new knowledge on how digital support for PA needs to be modified to fit the heterogeneous population of seniors. 

## Discussion

### Tracking of Subgroups’ Feedback in UCD

The method presented in this study provides a structured way to document and exploit feedback from different user subgroups in iterative development of new solutions, exemplified here by digital support systems for fall-preventive PA interventions in seniors. Involving seniors in technology development has been suggested as an important component for improving technology acceptance [10]. Moreover, physical inactivity is an increasing global health burden, and it is well documented that older age groups are less active than younger individuals [24] and that PA is important for improving health [9]. Moreover, although inclusiveness is an important design goal, discrimination is a common deficiency of digital support systems [14]. For example, gender inclusiveness and equality are important in system design, since the two aspects influence users’ behaviors, both online and offline [15,26]. GenderMag [30] is a systematic method for illustrating and tracking gender differences in software development by the use of personas. The method has proven effective for finding and fixing gender-inclusiveness deficiencies in software applications [31] and has been reported to be appreciated in practice [32]. Research on how to prevent discrimination and strengthen inclusiveness of digital support systems needs further attention.

In addition to increasing gender inclusion, the method presented in this study aims at ensuring that the feedback of users with varied self-reported technology experience and PA levels is shaping the development of digital support systems. The aim of integrating these perspectives is to encourage participation of persons who, because of lower levels of technology experience or PA, might have low interest in participating in UCD studies of technical solutions supporting PA. However, other user characteristics might also be of relevance when involving seniors in UCD studies. For example, Vandekerckhoven et al [11] suggested that creativity and communication are relevant and important user characteristics to consider in UCD studies.

### Limitations

In this study, the users’ gender, PA level, and level of technology experience were identified as relevant key user characteristics to define the subgroups to track in UCD studies. It could be argued whether these are the most critical key user characteristics to consider and if additional subgroups are to be included in order to support the development of digital support systems that fits the whole heterogenous target population. For example, fall history might be relevant to consider, since previous falls have been identified as a risk factor for new falls in both men and women [18] and it is vital to promote fall-preventive PA interventions in persons with increased fall risk. Moreover, users’ level of technology experience and internet use are related to age [16,33]: younger seniors (mean age less than 70 years) use the internet the most and older seniors (mean age 73 years) use the internet the least [16]. Furthermore, older seniors have more problems with activities in daily living and experience worse health than younger seniors [16]. However, this study used internet experience instead of age as a key user characteristic. To ensure that both younger and older seniors are involved in the UCD process, seniors representing different ages should be recruited.

Other examples of user characteristics that might be relevant for defining user subgroups include level of education and socioeconomic status [12,33], as well as personal qualities such as the level of communication and creativity [11]. However, in order to make the method feasible, the number of subgroups considered needs to be limited. This is accomplished by selecting user characteristics that are critical for the aim of the technical solution and can be dichotomized. In this study, 3 key user characteristics were selected based on the literature, and by dichotomizing them, 8 (ie, 2^3^) possible user subgroups were defined. The method represents a pragmatic approach, which is needed in UCD for balancing the quantitative research paradigm (requiring involvement of large user groups and thereby not feasible in UCD) and the qualitative research paradigm (focusing on one specific user subgroup and thereby not useful for an entire heterogenous population). However, further research is needed to validate if the key characteristics selected in this study are purposeful for creating a solution that is attractive and useful for the intended users of digital support systems for fall-preventive PA interventions for seniors.

### Conclusions

User involvement alone does not guarantee that feedback from different user subgroups is correctly shaping the development of digital support systems and resulting in a solution that is useful for the whole intended user population. Further attention is needed on methods for systematic tracking of user subgroups’ feedback to ensure that new systems and services are designed for the entire target user population. Also, new knowledge is needed on how to select users to be involved in UCD processes. The method presented in this study elucidates and documents potential differences between how different user subgroups contribute to the development. This may clarify whether new users need to be added to the process, either to increase the contribution of specific user subgroups or to involve new subgroups.

Our hope is that this protocol will be used for systematic analysis and prioritization of user subgroups’ feedback in the development of new digital systems. The protocol may help to identify and improve the understanding of potential differences between subgroups of seniors in use and experiences of digital support for fall-preventive PA interventions. This new knowledge can be of great importance in future research to develop systems that are relevant, useful, and attractive to the heterogenous population. Moreover, it can facilitate tailoring solutions toward specific user subgroups. However, the protocol needs to be further used and evaluated to validate the potential value of the method and the purposefulness of the selected key user characteristics.

## Appendix

Multimedia Appendix 1 Questionnaire on demographics, physical activity level, and level of technology experience to be completed by the participants.

Multimedia Appendix 2 Observation protocol to be completed by the researcher during the test.

Multimedia Appendix 3 Interview guides for the 4 test cycles in the usability test.

## Abbreviations

PA: physical activity
UCD: user-centered design
