# Supplementary material for: Integrating Key User Characteristics in User-Centered Design of Digital Support Systems for Seniors’ Physical Activity Interventions to Prevent Falls: Protocol for a Usability Study
Source: JMIR Res Protoc. 2020 Dec 21;9(12):e20061. doi: 10.2196/20061 (PMC7781794; doi:10.2196/20061)
Supplement: Multimedia Appendix 1 [file resprot_v9i12e20061_app1.docx]

| **Questionnaire on demographics, physical activity level and level of technology experience** |
| --- |
| **Demographics:**   1. Your gender   Man  Woman   1. How old are you?   ___________years   1. Do you need help with daily activities?   None  From family  From homecare   1. Do you use walking aids?   None  Use crutches outdoors  Use walker or crutches in-doors   1. How do you estimate your memory?   Good  Somewhat bad  Very bad   1. Have you had any of the following diseases or health problems during the last 12 months? (mark all relevant diseases in the list)   Heart disease  Diabetes  Depression  Cancer  Rheumatism  Neurological disease, e.g. Parkinson’s’ disease or stroke  Sight or hearing deficit that cannot be compensated  Bowel or intestinal disease   1. Have you used, or do you use any of the following medications during the last 2 weeks? (mark all relevant medications in the list)   Blood pressure  Diuretic  Sleeping pills  Sedative  Anti-depressive  Other_____________  **Physical activity level:**   1. How much time do you spend a regular week on performing moderate intensity activities that get you warm? (e.g. brisk walking, gardening, cycling)   Never  Less than 1 hour  Between 1-3 hours  More than 3 hours but less than 5  At least 5 hours   1. How active have you been the last 6 months?   Almost not at all  Mostly sedentary, sometimes a walk or gardening  Low intensity activities 2-4 hours per week, e.g. walking, dancing  Moderate intensity activities 1-2 hours per week, e.g. running, swimming, aerobics, or low activities more than 4 hours per week  Moderate intensity activities at least 3 hours per week, e.g. tennis, swimming, running  Regular high intensity exercises several times per week, e.g. running, skiing)  **Technology experience:**   1. Do you own a smart phone, computer, tablet?   Yes  No   1. Do you own a computer or tablet?   Yes  No   1. How often do you use a mobile phone?   Almost never  Use it often to make calls  Use it often for calls, text messaging and to surf on the internet   1. How often do you use a computer or tablet?   Almost never  Use it often, mostly for emails  Use it often for emails and to surf the internet   1. How would you estimate your general technology interest?   Not at all interested  Very interested |
