# Supplementary material for: Integrating Key User Characteristics in User-Centered Design of Digital Support Systems for Seniors’ Physical Activity Interventions to Prevent Falls: Protocol for a Usability Study
Source: JMIR Res Protoc. 2020 Dec 21;9(12):e20061. doi: 10.2196/20061 (PMC7781794; doi:10.2196/20061)
Supplement: Multimedia Appendix 2 [file resprot_v9i12e20061_app2.docx]

**Instructions:**

The researcher notes down the participant’s behavior during the test, e.g. actions, verbal expressions, difficulties that arise and how the participant solves the situation. The italic text provides additional explanation and a mock result for further clarification.

| **Observation protocol** | |
| --- | --- |
| **Participant no:** *01*  **Date:**  *4^th^ of May 2018*  **Test cycle no:** *2*  **Researcher:** *Åsa*  **Focus of the test cycle:** *Goal setting and planning of PA* | |
| **Description of the tasks to complete by the participant** | **Researcher’s notes** |
| 1. *Example: Log in to the behavior application* | Observed behavior:  *Takes time for P to find the button for capital letter*      Participant’s verbal expression:  *How do I change to capital letters…? Cannot find it.* |
| 1. *Description of task 2...etc.* | Observed behavior:      Participant’s verbal expressions: |
