# Supplementary material for: Integrating Key User Characteristics in User-Centered Design of Digital Support Systems for Seniors’ Physical Activity Interventions to Prevent Falls: Protocol for a Usability Study
Source: JMIR Res Protoc. 2020 Dec 21;9(12):e20061. doi: 10.2196/20061 (PMC7781794; doi:10.2196/20061)
Supplement: Multimedia Appendix 3 [file resprot_v9i12e20061_app3.docx]

| **Interview guide Test cycle 1**  *Focus area:* *Tablet and main menu user interface, Overall content and text sections in the behavior support application*  1. Please, tell me how it was for you to use the program  2. What do you perceive the program useful for? How do you believe the program could be useful for you?  3. How would you prefer to get support to enable you to use the program?  4. Do you have any other experiences or ideas you want to share with me?  5. How interesting is the texts about physical activity for you?  Very interesting  Not at all interesting       1. Did the texts evoke any feeling or thoughts?   A lot  Non    7. Do you have any questions or thoughts about the texts?  **Interview guide Test cycle 2**  *Focus area Part 1: Goal set in the behavior support application*  1. Please tell me how it was for you to use the application and to set goals?  2. Can you describe any difficulties you experienced when using the program.  3. How did you experience the help texts? (useful or not useful)  4. The application is now programmed for the user to be rewarded every week when activity goal is achieved. How often do you believe you would like to get rewarded?  6. How could the goal set functionality be improved according to you?  7. Do you have other experiences or ideas you want to share with me?  8. How important do you believe it is for you to set long term goals that motivates you to activity? E.g. to be able to play with my grandchildren, to go to the store back and forth, to improve blood pressure to X.  Very important  Not at all important      9. How important do you believe it is for you to set an activity and/or exercise goal? E.g. go to the gym 2 times per week, walk 20 minutes every day.  Very important  Not at all important    *Focus area Part 2: Calendar in the social support application*  1. Please tell me how it was for you to use the program and plan activity in the calendar?  2. Can you describe any difficulties you experience when using the program.  3. Tell me about your thoughts on getting reminders. What type of reminders would you prefer?  4. Can you tell me about what feedback you believe are important that the program provides for you to motivate physical activity? Suggestions?  5. How could the calendar be improved, according to you?  6. Do you have any other experiences or ideas you want to share with me?  7. How important is it for you to be able to plan activities and exercises in a calendar?  Very important  Not at all important      **Interview guide Test cycle 3**  *Part 1: Goal set in the behavior support application*  1. Please tell me how it was for you to use the program and to set goals?  2. Can you describe any difficulties you experienced when using the program.  3. Tell me how it was for you to estimate the time you spend on activities per day?  4. How did you experience the help texts? (useful or not useful)  5. How could the goal set functionality be improved according to you?  6. Do you have any other experiences and ideas you want to share with me?  7. How important is it for you to be rewarded?  Very important  Not at all important    8. What kind of rewards are important for you? Give some examples  *Part 2: Activity planning in the behavior support application*  1. Tell me how it was for you to plan an activity in the calendar?  2. Please, describe any difficulties you experienced when using the program.  3. How could the activity planning be improved according to you?  4. Do you have any other experiences and ideas you want to share with me?  5. How important do you believe it is for you to evaluate your performance?  Very important  Not at all important      6. How often do you want to do an evaluation? How do you want to evaluate?  **Interview guide Test cycle 4**  *Part 1: Goal set in the behavior support application*  1. Please tell me how it was for you to use the program and to set goals?  2. Can you tell me what you think about the welcome page?  3. Please, describe any difficulties you experienced when using the program.  4. What do you think about having a step counter integrated in the program and to enable you to set a goal for steps per day?  5. How could the goal set functionality be improved, according to you?  6. Do you have any other experiences or ideas you want to share with me?  7. What is your overall impression of the goal set functionality, i.e. to set a goal for activities and a goal for step per day?  Very easy to understand and use  Not at all easy to understand and use      *Part 2: Video call in the social support application*  1. Tell me how it was for you to use the videocall app:   - - With support?   - Without support?   - Get a call?   - Make a call to the coach?   2. Please, describe any difficulties you experienced when using the program  3. Can you tell me about any ideas or thoughts you have on how this video app could be used?  4. Do you have any other experiences or ideas you want to share with me?  5. How important is it for you to be able to get support on activity and exercise from someone professional (e.g. a physiotherapist)  Very important  Not at all important    6. Please, can you elaborate on your rating? |
| --- |
